# Supplementary material for: Complexome profiling of the Chlamydomonas psb28 mutant reveals TEF5 as an early PSII assembly factor
Source: Plant Cell. 2025 Mar 17;37(6):koaf055. doi: 10.1093/plcell/koaf055 (PMC12164590; doi:10.1093/plcell/koaf055)
Supplement: koaf055_Supplementary_Data [file koaf055_supplementary_data.zip › psb28_interactive.zip!/plots/intensity/qualityPlots/peptides/_Cre11.g467597.t1.1.html]

### Cre11.g467597.t1.1

Trivial name:   
SeqCoverage [median]: 10.40  
Mass: 159.88 kDa  
  
 

| Sequence | MissedCleavage | Mass [Da] | wtIntensity | muIntensity | Score |
| --- | --- | --- | --- | --- | --- |
| ALGVSMPSVTLEYR | 0 | 1521.79 | 0.00 | 2288.34 | 94.82 |
| AMGIAHTANTPVGDAMLR | 0 | 1824.90 | 387.30 | 1148.28 | 82.71 |
| EAFALQENDLATR | 0 | 1476.72 | 396.05 | 1531.94 | 152.85 |
| GGGFDLPGELRR | 1 | 1272.66 | 0.00 | 1974.98 | 72.65 |
| LGLPRPGAAATPGVSSPPSQSK | 1 | 2074.12 | 0.00 | 1831.30 | 69.12 |
| LLEQPFDK | 0 | 988.52 | 0.00 | 785.62 | 68.42 |
| LLEQPFDKAK | 1 | 1187.65 | 0.00 | 849.78 | 45.16 |
| VPDPDELLAAR | 0 | 1194.62 | 667.86 | 0.00 | 65.31 |
| VQAADEQLKEYGGFTAAMGAR | 1 | 2212.06 | 0.00 | 1137.15 | 87.33 |
| VTTAEMLVGPR | 0 | 1172.62 | 0.00 | 951.85 | 108.98 |
| YFVPNPSAGVK | 0 | 1177.61 | 0.00 | 2232.50 | 76.83 |
|  |  |  | 1451.2 | 14731.7 |  |
